# Recipient Age Predicts 20-Year Survival in Pediatric Liver Transplant

**DOI:** 10.1155/2022/1466602

**Published:** 2022-09-17

**Authors:** Stephanie Keeling, Malcolm F. McDonald, Adrish Anand, Jordan Dunson, Elizabeth Williams, Theodore Zhang, Brian Hickner, Nhu Thao Nguyen Galván, Christine O' Mahony, John A Goss, Abbas Rana

**Affiliations:** ^1^Department of Student Affairs, Baylor College of Medicine, Department of Medicine, Houston 77030, TX, USA; ^2^Division of Abdominal Transplant, Michael E DeBakey Department of Surgery, Baylor College of Medicine, Houston 77030, TX, USA

## Abstract

**Introduction:**

Pediatric liver transplant recipients have demonstrated excellent long-term survival. The purpose of this analysis is to investigate factors associated with 20-year survival to identify areas for improvement in patient care.

**Methods:**

Kaplan–Meier with log-rank test as well as univariate and multivariate logistic regression methods were used to retrospectively analyze 4,312 liver transplant recipients under the age of 18 between September 30, 1987 and March 9, 1998. Our primary endpoint was 20-year survival among one-year survival.

**Results:**

Logistic regression analysis identified recipient age as a significant risk factor, with recipients below 5 years old having a higher 20-year survival rate (*p* < 0.001). A preoperative primary diagnosis of a metabolic dysfunction was found to be protective compared to other diagnoses (OR 1.64, CI 1.20–2.25). African-American ethnicity (OR 0.71, CI 0.58–0.87) was also found to be a risk factor for mortality. Technical variant allografts (neither living donor nor cadaveric) were not associated with increased or decreased rates of 20-year survival.

**Conclusions:**

Our analysis suggests that long-term survival is inversely correlated with recipient age following pediatric liver transplant. If validated with further studies, this conclusion may have profound implications on the timing of pediatric liver transplantation.

## 1. Introduction

Orthotopic liver transplantations have been a life-saving treatment for irreversible liver disease since the first human liver transplant in 1963. [1] Pediatric liver transplantation is a durable treatment with excellent long-term survival, often decades post-transplant. [2] In recent years, the rates of liver disease have steadily risen without a corresponding surge in liver donations. [3] Specifically, there has been an increase in both the number of pediatric transplant patients and in the severity of their illnesses. [4–6] As liver transplants are becoming an increasingly rare commodity, a need arises to optimize treatment for each patient using objective evidence-based criteria and to investigate with greater scrutiny the factors contributing to long-term survival of pediatric liver transplant recipients.

A 10-year follow-up of pediatric liver transplant recipients has reported recipient and graft survival rates of over 90% and 85%, respectively. [7] Similar studies have quantified long-term patient survival for adult patients at 60% at 10-year follow-up. [8] The discrepancy in these survival rates has been theorized to result from a lack of unrelated comorbidities in the pediatric population, to which an estimated half of deaths in the adult population is attributed. [9]

Despite these much higher rates of long-term survival, pediatric liver transplant recipients are not exempt from complications. Late deaths in this population are commonly a result of immunosuppression rather than unrelated illness in the adult population. [9] As such, graft rejection resulting in retransplant, post-transplant lymphoproliferative disorder (PTLD), and infection account for most of these late deaths. [10].

Identifying factors that contribute to long-term survival outcomes in the pediatric population is crucial to the goal of ensuring the most effective use of the donated organ and reducing morbidity and mortality in the recipient. Attempts have been made to create a model that utilizes preoperative risk factors to assess the likelihood of long-term survival in the adult population, with some showing greater success than the Model for End-Stage Liver Disease (MELD) at predicting postoperative survival. [3, 11, 12] These indices cite factors such as recipient age, bilirubin, and cold ischemia time as significant for patient survival; however, these indices demonstrate little utility in the scope of pediatric liver transplantation. [12]

Although pediatric liver transplantation remains a reliable treatment for a variety of hepatobiliary pathologies, elucidating the factors that contribute most to long-term survival is necessary. [2] This analysis investigates these factors to identify areas for improvement in long-term outcomes for the pediatric liver transplant population.

## 2. Materials and Methods

### 2.1. Study Population

We retrospectively analyzed de-identified patient data from all recipients of donor livers under the age of 18 between September 30, 1987 and March 9, 1998 using the United Network for Organ Sharing (UNOS). Only donor and recipient characteristics reported at the time of transplant were included in the analysis. Patients were excluded from analysis if they underwent multivisceral transplantation (*n* = 1471). Patients who were deceased within one year from their transplant were also excluded from analysis (*n* = 1681). This is an accepted methodology to assess long-term survival as it removes the confounding impact of short-term mortality. [13] Significant gains or losses in short-term mortality may skew long-term survivor outcomes, thus, dropping deaths occurring within the first year removes this potential influence. [13] Data were also analyzed without excluding deaths within one year (*n* = 5197, Supplementary Table 1 (Table S1), Supplementary Figure 1 (Figure S1)). A total of 4,312 patients received a liver transplant during the study period. Patients were followed until death (*n* = 513) or last known follow-up (*n* = 3799).

### 2.2. Institutional Review Board Approval

Institutional Review Board (IRB) approval was obtained prior to the analysis of data and drafting of the manuscript. All patient data used in the drafting of this manuscript has been de-identified to preserve patient confidentiality.

### 2.3. Statistical Analysis

Data were analyzed using a standard statistical software package, Stata® 13.0 (Stata Corp, College Station, TX). Continuous variables were reported as mean ± standard deviation and compared using the Student's *t*-test. Contingency table analysis was used to compare categorical variables. Results were considered significant at a *p*-value of <0.05 and all reported *p*-values were two-sided.

Post-transplant survival analysis was performed using Kaplan–Meier with log-rank test as well as univariate and multivariate logistic regression methods. Covariates that were not significant (*p* > 0.05) were removed using stepwise backwards elimination Cox regression analysis. Primary outcome was defined as survival of 20 years or more after the transplant date as reported by UNOS. Contingency table analysis was used to compare survival outcomes at 20 years after UNOS reported transplant dates. Cox-regression analysis was then used once groups were stratified into three groups based on the presence of protective and risk factors.

### 2.4. Risk Factors

The recipient risk factors considered in this analysis were age, weight, primary diagnosis, blood type, albumin, creatinine, hemodialysis status, presence of Status 1 criteria, ethnicity, recipient transplant history, intensive care unit (ICU) stay, hospital admission, life support status, glomerular filtration rate (GFR), total bilirubin, ascites, bacterial peritonitis, BMI, encephalopathy, ventilation status of recipient, private insurance, previous abdominal surgery, and wait list time. The donor risk factors considered in this analysis were age, ethnicity, cause of death, live donor transplant, split transplant, cold-ischemia time, warm ischemia time, total bilirubin, liver function tests, diabetes mellitus (DM) status, blood type compatibility, distance of allograft travel, height difference between recipient and donor, weight difference between recipient and donor, and portal vein transplant. Creatinine clearance was calculated with the updated Schwartz bedside formula: eGFR = 0.41 X height (cm)/Scr (mg/dL). Pediatric End-stage Liver Disease (PELD) scores, Model for End-Stage Liver Disease (MELD) scores, and serum sodium were not used because they were not reliably recorded before 2002. Missing variables were imputed using the predictive mean matching imputation method for incomplete data entry in the UNOS database. [13]

## 3. Results

### 3.1. Study Population

**Figure 1:**
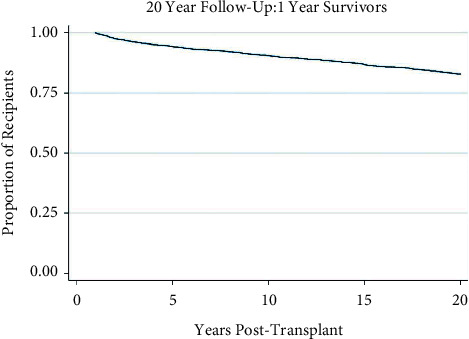
Kaplan–Meier survival function over time for 1-year survivor pediatric liver transplant recipients.

From 1987 to 1998, the study population consisted of 4,312 patients, which consists of 1307.8 years-at-risk. Demographic and clinical characteristics are summarized in Table 1. Multivariate logistic regression analysis of allograft, donor, and recipient factors is summarized in Table 2. Factors significant for 20-year survival were recipient age, donor age, primary diagnosis of metabolic dysfunction, primary diagnosis of biliary atresia, transplant history, and African-American ethnicity.  Figure 1 illustrates the Kaplan– Meier survival curve of the study population.

### 3.2. Data Entry Rate

The percentages for data entry by variable are summarized in Table 2. Completion percentages for most variables exceeded 90%. Missing variables were imputed using the predictive mean matching imputation method.

### 3.3. Recipient and Donor Age

**Figure 2:**
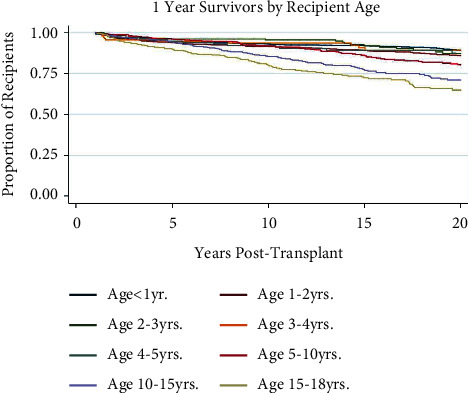
Kaplan–Meier survival function over time for 1-year survivor pediatric liver transplant recipients by recipient.

**Figure 3:**
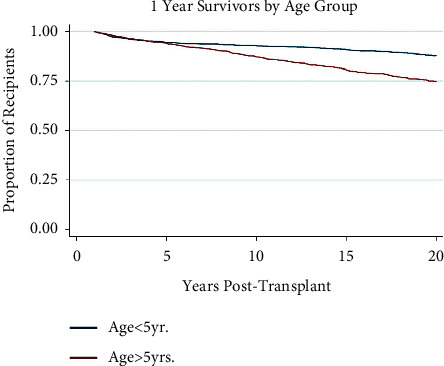
Kaplan–Meier survival function over time for 1-year survivor pediatric liver transplant recipients by recipient age group.

Table 1 demonstrates that both recipient and donor age are significantly younger in the 20-year survival population (*p*-value <0.001). Multivariate logistic regression analysis supports longer survival for younger recipient ages at transplant (Table 2). Although each recipient age stratification is not statistically significant, analysis suggests an overall trend toward early mortality in older recipients.  Figure 2 demonstrates this trend with older recipients having worse outcomes at 20 years post-transplant, beginning with the 5–10-year-old cohort.  Figure 3 illustrates this trend, with those younger than 5 years having significantly higher rates of survival at 20 years post-transplant at 87.8% and those older than 5 having a rate of 74.8% (*p* < 0.001). Younger donor age also seems to be a protective factor, trending toward risk as age increases (Table 2).

### 3.4. Primary Recipient Diagnosis

**Figure 4:**
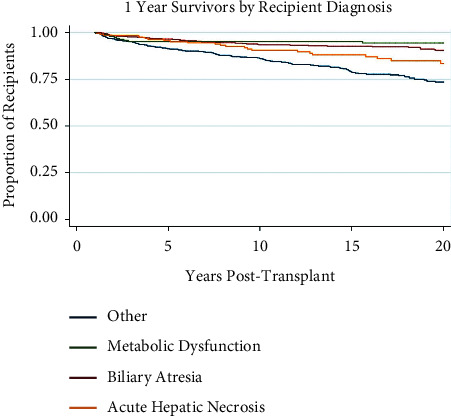
Kaplan–Meier survival function over time for 1-year survivor pediatric liver transplant recipients by primary recipient diagnosis.

A significantly greater percentage of patients with biliary atresia and metabolic dysfunction were present in the 20-year survival population (Table 1; *p*-value < 0.001). Diagnosis of metabolic dysfunction is protective (Odds Ratio (OR) 1.64 (1.20–2.25) for 20-year survival (Table 2).  Figure 4 illustrates Kaplan–Meier survival curves for the four most common primary recipient diagnoses as reported in UNOS, which are biliary atresia (*n* = 643), metabolic dysfunction (*n* = 201), acute hepatic necrosis (*n* = 218), and other (*n* = 3070). Survival of recipients with a primary diagnosis of metabolic dysfunction or biliary atresia is significantly greater at 20 years post-transplant than other diagnoses (*p* < 0.001). This survival benefit for biliary atresia was not replicated in the adjusted multivariate analysis; biliary atresia was not found to be a significant protective or risk factor (*p*-value = 0.675).

### 3.5. Recipient Ethnicity

**Figure 5:**
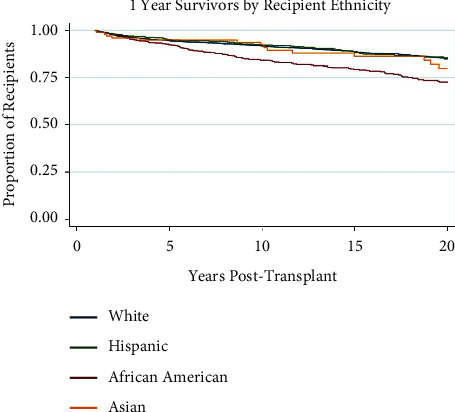
Kaplan–Meier survival function over time for 1-year survivor pediatric liver transplant recipients by recipient ethnicity.

African-American recipients have greater rates of mortality (OR 0.71 (0.58–0.87) than other ethnic groups (Table 2).  Figure 5 illustrates the Kaplan–Meier survival curve for the most commonly reported ethnic groups in the UNOS database, being white (*n* = 2729), African-American (*n* = 742), Hispanic (*n* = 628), and Asian (*n* = 123). African-American recipients had the lowest overall survival at 20 years post-transplant at 72.7%, which was significant at *p* < 0.001 when compared to all other ethnic groups.

### 3.6. Recipient Transplant History

**Figure 6:**
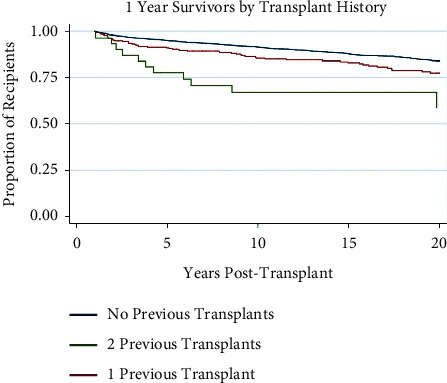
Kaplan–Meier survival function over time for 1-year survivor pediatric liver transplant recipients by recipient transplant history.

As expected, previous transplants were inversely correlated with long-term survival. Recipients with 1 previous transplant OR 0.74 (0.57–0.94) and 2 previous transplants OR 0.37 (0.16–0.82) had progressively worse survival rates at 20 years post-transplant, respectively (Table 2).  Figure 6 illustrates this trend toward higher rates of mortality with greater number of previous transplants. Patients with 1 previous transplant (*n* = 201) and 2 previous transplants (*n* = 29) had significantly higher rates of mortality at 20 years post-transplant (*p* < 0.001) than recipients with no previous transplants (*n* = 1541).

### 3.7. Risk Factor Stratification

**Figure 7:**
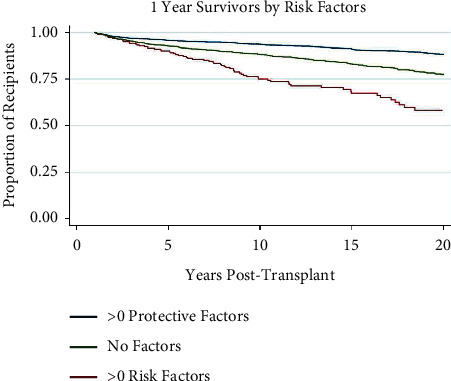
Kaplan–Meier survival function over time for 1-year survivor pediatric liver transplant recipients by risk factors.

Recipients were stratified into three groups based on the number of significant risk factors or protective factors.  Table 3 illustrates the results of the Cox regression analysis for each of these groups, which are >1 protective factor (*n* = 1139, HR 0.43, CI 0.32–0.58), no risk or protective factors (*n* = 328, HR 0.99, CI 0.87–1.12), and >1 risk factor (*n* = 664, HR 1.30, CI 1.16–1.46).  Figure 7 demonstrates the Kaplan–Meier survival curves for each of these groups. There were significantly greater rates of mortality in the risk factor group at 20 years post-transplant (*p* < 0.001). The protective factor group demonstrated significantly higher rates of survival at 20 years post-transplant (*p* < 0.001).

### 3.8. Supplementary Data: Analysis of Data with All Survivors

Data were also analyzed without excluding patients who died within one year of transplant (*n* = 5197). Similar trends were observed in this cohort of patients as were seen in the cohort which only included 1-year survivors (*n* = 4312). Multivariate logistic regression analysis is summarized in Table S1. Kaplan–Meier survival function for this cohort graphed by recipient age appears in figure S1.

## 4. Discussion

This analysis found that a significant risk factor for post-transplant mortality was the age of the recipient and donor at the time of the transplant, primary recipient diagnosis, recipient transplant history, and recipient ethnicity. Younger recipients had decreased 20-year mortality along with patients with primary diagnoses of metabolic dysfunction and biliary atresia. African-American recipients, recipients with one or more previous transplants, and recipients with other diagnoses had worse 20-year survival rates.

Many of these risk factors that contribute to long-term survival outcomes have been well studied in the adults but remain poorly understood in the pediatric population. We were thus able to fill this gap in the literature and better understand the impact of risk factors on long-term survival with the goal of maximizing the utility of the supply of organs available for transplant while prioritizing outcomes for individual pediatric patients in the future.

Twenty-year post-transplant survival was found to be inversely correlated with recipient age. More specifically, patients under 5 years of age at the time of transplant had a significantly greater 20-year survival rate than those between the ages of 10 and 15. This finding is reflective of an overarching trend in transplant medicine. Recipient age as a risk factor for long-term post-transplant survival has been widely studied in adult populations, although our analysis in the pediatric population was not expected to mirror this trend. [8, 14, 15] There is a variety of proposed explanations for this trend; older patients are more likely to suffer from cardiovascular disease, develop chronic kidney disease, and the rate of extrahepatic malignancies directly increase with age. [8, 15, 16] It could be reasoned that over the course of the following 20 years, the older recipients in our study had a greater likelihood of developing age-related maladies associated with mortality. Additionally, more naïve immune systems in younger patients may be able to accept an allograft more readily than an older patient and thus had a lower risk of developing graft rejection. Literature assessing long-term survival in pediatric kidney transplantation identifies adolescence as a risk factor. Pediatric patients transplanted at an older age will reach that risk period earlier, facing the known development risk for medication nonadherence closer to the time of transplant. [17] Age as a risk factor for transplant survival has not been well-studied in pediatric populations with liver disease, although our data supports that a younger age at the time of transplant correlates with greater 20-year survival rates. This finding may contradict the common clinical decision to delay transplantation in children for the benefit of allowing a child to live without the burden of transplant and allowing the child to mature physically before such a major procedure; however, further investigation is required prior to such a drastic shift in clinical decision-making.

One possible explanation for worse outcomes for older recipients is medication nonadherence. Medication nonadherence has been reported to be a leading cause of late mortality and graft failure, especially in recipients in their teenage years. [9] While this is a reasonable consideration, we might expect medication noncompliance to appear as sharp declines in survival curves at predictable times. For example, a cohort transplanted at less than one year of age would be expected to show declines in survivorship beginning at 13–18 years post-transplant, the time at which they are expected to have the least compliance. [9] Instead, we see gradual declines in survival as recipient age increases, possibly indicating that increased age poses inherent risk to long-term survival. As mentioned above, greater rates of comorbidities and less flexible immune systems may be a more suitable explanation for the observed trend.

A related trend is that of donor age as a risk factor impacting post-transplant survival. Liver function does not significantly decrease with age, and yet prior research has found that livers from older donors carry a greater risk of graft failure and are correlated with worse long-term outcomes for adult recipients. [8, 15, 16] Survival is lower in those who receive an organ from an older donor. [18–22] The pathophysiological basis of this association is not well established, but different biological changes in aging could lead to a loss of the liver's proliferative response and regeneration. [18] Our results echo these findings in a pediatric population, as donor age was found consistently to be negatively correlated with survival across all cohorts. Additionally, the mortality risk increased as the age difference between the donor and the recipient grew. This finding is particularly relevant in the scope of modern transplantation as the growing demand for organs necessitates the use of older donors. [8] In fact, the age of liver donors has been steadily increasing in the United States, with over 33% of donors now being over 50 years of age compared to 1.5% of donors prior to 1985. [8, 9, 15–18].

A particularly interesting finding of our analysis was the role of primary recipient diagnosis as a risk factor for survival. A primary diagnosis of metabolic dysfunction was protective and associated with a greater 20-year survival rate. Metabolic dysfunction is the second most common indication for liver transplants in pediatric populations, only falling behind biliary atresia. [23–27] It is well documented that this primary diagnosis is correlated with post-transplant survival. [9, 15–27] Our results confirm prior findings that pediatric patients receiving a liver transplant as treatment for a metabolic disease have greater short- and long-term survival than those receiving a transplant for other diagnoses. [15, 18, 23, 26, 28] The reasons for this protective status are many, with a significant reason being that in many metabolic liver diseases, a liver transplant is not only a treatment but a cure to the underlying disease. [23] For example, in *α*‐1‐antitrypsin deficiency, liver transplantation allows for complete amelioration of the underlying metabolic defect, and in Wilson's disease, a liver transplant not only cures the underlying disorder, but it also reverses the neurological manifestations of the disorder. [23, 29] Such may also be the case for patients with a diagnosis of biliary atresia. The curative nature of transplant in these patients eliminates disease burden, thus improving long-term survival rates.

Technical variant liver transplant techniques (including split, live-donor, and reduced allografts) were created to meet the increasing demand for liver allografts. Contention has arisen surrounding potential risk that these procedures carry. [30] Specifically, technical variant outcomes have been previously documented to have worse outcomes compared to whole-organ recipients, including perioperative complications and recipient mortality. [30, 31] Our analysis indicates that technical variant allografts bear no additional risk or benefit for 20-year survival compared to whole-organ transplantation. This finding may coincide with other studies indicating that much of the mortality associated with technical variant allografts occurs in the perioperative period. [32] Our analysis excluded deaths occurring within 1-year of transplantation, which may not have captured increased morbidity and mortality in the perioperative period. [32] Nevertheless, it can be surmised from our analysis that long-term survival is unaffected by the usage of technical variant allografts. These findings support the use of technical variant allografts, which in turn, would increase the number of available allografts to meet high demand [30–32].

The disparity in transplant survival across ethnicities has been well documented in adults. [33–38] However, the continuation of this trend into the realm of pediatrics is a subject of some debate. Some research has found that the racial disparity in transplant survival exclusively exists in adult populations, [39] while other studies propose the inequality permeates into pediatrics and specifically applies to pediatric liver transplant recipients. [40, 41] A point of universal consensus is that African-Americans have worse transplant survival outcomes than other ethnicities. [27, 32–39, 41–44] Our research found that pediatric African-American liver transplant recipients have greater rates of mortality than other ethnic groups. The reasons for this inequality are far-reaching and involve every step of the transplantation process. African-Americans are less likely to be referred for a liver transplant evaluation, and their referral is more likely to be delayed. [35, 45] African-American patients also have higher average MELD scores at the time of transplantation listing. [34, 36, 46] Complications such as acute rejection and hepatic encephalopathy are recorded more frequently in African-American transplant recipients. [34, 37, 38, 45] All of these factors, along with pre-existing inequalities in access to healthcare and insurance coverage, combine to make ethnicity, specifically being African-American, a significant risk factor for post-transplant survival.

We project that a limitation of our study is the inability to analyze data within the modern era of transplantation. With UNOS data reaching until 2018, recipients with transplant date after 1998 were unable to be analyzed as 20 years had not elapsed since their transplant date. This limitation could hinder the analysis as recipient and donor risk factors could fluctuate over time. Despite this, our study has strength in study population size and exclusion of deaths within 1 year of transplant. Such exclusion allowed more accurate long-term survival analysis without the influence of patients with early mortality. In addition, since the registry used in this study only records variables at time of listing, time of transplant, and scheduled follow-ups until death, specific data related to clinical events such as rejections, infections, and complications may be unreliable. A better understanding of postoperative events could better elucidate reasons behind disparities in long-term survival observed in this study.

## 5. Conclusions

Our analysis demonstrates that long-term survival is inversely correlated with recipient age following pediatric liver transplant. Additionally, a primary recipient diagnosis of metabolic dysfunction was correlated with higher rates of survival across the 20-year study period. No significant difference in 20-year survival was found in technical variant allografts, providing support for the use of these allografts to enhance the donor pool. Lastly, our analysis showed that African-American recipients have greater rates of mortality than other ethnic groups. This highlights the need for increased support and vigilance for disparity in this population of patients.

If validated with further studies, this conclusion may have profound implications on the timing of pediatric liver transplantation. Identifying factors that contribute to long-term survival outcomes in the pediatric population is crucial to the goal of ensuring the most effective use of the donated organ. Elucidating these factors is necessary for improving outcomes in the pediatric liver transplant population. Further analysis of the impact of age on long-term post-transplant survival is necessary in reducing morbidity and mortality in pediatric recipients while ensuring the most effective use of the donated organ.

## Figures and Tables

**Table 1:** Demographics and clinical characteristics of allografts and recipients of 1-year survivors.

	Survival <20 years	Survival >20 years	*P*-value
Total recipients	3144	1168	NA

*Recipient demographics and characteristics*			
Recipient age	5.74 ± 5.99	4.01 ± 5.20	<0.001
Weight (kg)	23.40 ± 21.61	17.54 ± 18.21	<0.001
Diagnosis: biliary atresia	432 (13.74%)	211 (18.11%)	<0.001
Diagnosis: metabolic dysfunction	126 (4.01%)	75 (6.42%)	<0.001
Albumin (mg/dL)	3.17 ± 0.70	3.21 ± 0.69	0.072
Creatinine	0.60 ± 0.82	0.50 ± 0.80	<0.001
Hemodialysis at transplant	34 (1.08%)	13 (1.11%)	0.929
No previous transplants	2430 (77.29%)	986 (84.42%)	<0.001
1 previous transplant	362 (11.51%)	97 (8.30%)	0.002
2 previous transplants	51 (1.62%)	7 (0.60%)	0.010
Intensive care at transplant	1017 (32.35%)	305 (26.11%)	<0.001
Life support at transplant	616 (19.59%)	174 (14.90%)	<0.001
Recipient total bilirubin	12.95 ± 11.93	12.11 ± 11.83	0.041
Ascites	692 (22.01%)	269 (23.03%)	0.474
BMI	18.88 ± 9.91	18.04 ± 9.27	0.022
Encephalopathy at transplant	200 (6.36%)	64 (5.50%)	0.283
African-American recipient	585 (18.60%)	157 (13.44%)	<0.001
Ventilation at transplant	582 (18.51%)	161 (13.78%)	<0.001
Private insurance	688 (21.88%)	290 (23.03%)	0.040
Previous abdominal surgery	666 (21.18%)	293 (25.10%)	0.006
Time listed before transplant (Years)	0.32 ± 0.54	0.36 ± 0.63	0.051

*Donor and allograft demographics and characteristics*			
Donor age	14.15 ± 15.00	10.14 ± 12.11	<0.001
African-American donor	430 (13.68%)	152 (13.01%)	0.571
Cause of death: cerebrovascular accident	506 (16.09%)	142 (12.16%)	<0.001
Cause of death: deceased cardiac donor	2 (0.06%)	2 (0.17%)	0.022
Live donor transplant	247 (7.86%)	76 (6.51%)	0.135
Split transplant	386 (12.28%)	156 (13.36%)	0.342
Cold ischemia time (hours)	10.57 ± 5.90	10.73 ± 6.31	0.437
Donor total bilirubin	0.99 ± 2.63	0.99 ± 2.62	0.71
Donor: diabetes mellitus	17 (0.54%)	4 (0.34%)	0.406

**Table 2:** Multivariate logistic regression for factors that predict 20-year survival in 1-year survivors.

	Entry completion (%)	OR	*P*-value
Age <1	100%	1.28 (1.07–1.53)	0.007
Age 5–10	100%	0.86 (0.67–1.10)	0.231
Age 10–15	100%	0.72 (0.51–0.99)	0.046
Age 15–18	100%	0.82 (0.57–1.20)	0.307
Donor age <1	99%	1.27 (1.00–1.61)	0.048
Donor age 1–2	99%	1.25 (1.01–1.54)	0.042
Donor age 2–3	99%	1.49 (1.10–2.01)	0.009
Donor age 3–4	99%	1.85 (1.35–2.53)	<0.001
Donor age 5–10	99%	1.57 (1.28–1.93)	<0.001
Weight 4–12 kg	94%	1.17 (0.96–1.43)	0.129
Weight >30 kg	94%	0.96 (0.74–1.23)	0.727
Diagnosis: biliary atresia	100%	1.05 (0.83–1.33)	0.675
Diagnosis: metabolic dysfunction	100%	1.64 (1.20–2.25)	0.002
Creatinine 1.5–2.0	98%	0.75 (0.40–1.39)	0.359
Creatinine >2.0	98%	0.83 (0.52–1.33)	0.448
Donor cause of death: CVA	92%	0.86 (0.70–1.06)	0.153
Cold ischemia time > 4 hrs	89%	1.31 (0.98–1.75)	0.065
Cold ischemia time 10–12 hrs	89%	1.16 (0.97–1.38)	0.095
1 previous transplant	91%	0.74 (0.57–0.94)	0.016
2 previous transplants	91%	0.37 (0.16–0.82)	0.015
ICU at transplant	99%	0.91 (0.73–1.12)	0.367
Life support at transplant	100%	1.23 (0.62–2.45)	0.561
Recipient total bilirubin >33 mg/dL	98%	0.84 (0.65–1.09)	0.197
Height difference >30 cm	55%	0.59 (0.32–1.08)	0.087
African-American recipient	100%	0.71 (0.58–0.87)	0.001
Ventilator at transplant	100%	0.76 (0.38–1.51)	0.431
Privately insured	43%	1.11 (0.93–1.33)	0.248
Previous abdominal surgery	99%	1.12 (0.91–1.38)	0.273

**Table 3:** Multivariable cox regression for risk stratification groups: Hazard ratios for mortality.

	Hazard Ratio	95% Confidence Interval
>1 protective factor	0.43	0.32–0.58
No factors	0.99	0.87–1.12
>1 risk factor	1.30	1.16–1.46

## Data Availability

The data that support the findings of this study are available in the Scientific Registry of Transplant Recipients (SRTR) database which can be requested from SRTR at https://www.srtr.org.

## Abbreviations:

(DM): Diabetes mellitus
(GFR): Glomerular filtration rate
(ICU): Intensive care unit
(MELD): Model for end-stage liver disease
(PELD): Pediatric end-stage liver disease
(PTLD): Post-transplant lymphoproliferative disorder.
(UNOS): United network for organ Sharing

## Additional Points

This study was approved by the institutional review board.

## Supplementary Materials

Supplementary Materials Supplementary Materials Table S1: multivariate logistic regression for factors that predict 20-year survival in all survivors. Figure S1: Kaplan–Meier survival function over time for all survivors in pediatric liver transplant recipients by recipient age.
