# Supplementary material for: Recipient Age Predicts 20-Year Survival in Pediatric Liver Transplant
Source: Can J Gastroenterol Hepatol. 2022 Sep 17;2022:1466602. doi: 10.1155/2022/1466602 (PMC9509270; doi:10.1155/2022/1466602)
Supplement: Supplementary Materials — Table S1: multivariate logistic regression for factors that predict 20-year survival in all survivors. Figure S1: Kaplan–Meier survival function over time for all survivors in pediatric liver transplant recipients by recipient age. [file 1466602.f1.docx]

| **Table S1. Multivariate Logistic Regression for Factors that Predict 20-Year Survival in All Survivors** | | | |
| --- | --- | --- | --- |
|  | **Entry Completion (%)** | **OR** | **P-Value** |
| **Age 4-5 Years** | **100%** | **1.56 (1.09-2.24)** | **0.015** |
| **Age 10-15** | 100% | 0.82 (0.60-1.10) | 0.188 |
| Age 15-18 | **100%** | 1.02 (0.72-1.44) | 0.93 |
| **Donor Age < 1** | 99% | 1.22 (0.96-1.55) | 0.108 |
| **Donor Age 1-2** | **99%** | 1.18 (0.95-1.48) | 0.134 |
| **Donor Age 2-3** | **99%** | **1.41 (1.04-1.91)** | **0.026** |
| **Donor Age 3-4** | **99%** | **1.61 (1.17-2.21)** | **0.003** |
| **Donor age 5-10** | **99%** | **1.46 (1.18-1.81)** | **<0.001** |
| **Weight 4-12kg** | **99%** | **1.28 (1.07-1.53)** | **0.007** |
| Weight > 30kg | 99% | 0.95 (0.74-1.23) | 0.71 |
| Diagnosis: Biliary Atresia | 100% | 2.07 (1.74-2.46) | **<0.001** |
| **Diagnosis: Metabolic Dysfunction** | 100% | 2.02 (1.66-2.46) | **<0.001** |
| Creatinine 1.5-2.0 | **98%** | 0.62 (0.34-1.14) | 0.124 |
| Creatinine > 2.0 | 98% | 0.67 (0.42-1.05) | 0.082 |
| Donor Cause of Death: CVA | 92% | 0.85 (0.69-1.04) | 0.113 |
| **Cold Ischemia Time > 16hrs** | **88%** | **0.74 (0.62-0.89)** | **0.001** |
| **1 Previous Transplant** | 91% | 0.64 (0.50-0.81) | **<0.001** |
| **2 Previous Transplants** | **91%** | **0.27 (0.12-0.59)** | **0.001** |
| ICU at Transplant | **99%** | 0.96 (0.77-1.19) | 0.682 |
| Life Support at Transplant | 100% | 1.18 (0.61-2.28) | 0.627 |
| Donor Total Bilirubin > 1.8mg/dL | 34% | 0.99 (0.82-1.19) | 0.908 |
| Recipient Total Bilirubin > 33mg/dL | 98% | 0.78 (0.60-1.00) | 0.053 |
| Recipient Encephalopathy at Transplant | 100% | 1.24 (0.90-1.70) | 0.19 |
| **African American Recipient** | 100% | 0.70 (0.57-0.85) | **<0.001** |
| Ventilator at Transplant | 100% | 0.61 (0.31-1.17) | 0.136 |
| Privately Insured | 42% | 1.07 (0.89-1.29) | 0.452 |
| Previous Abdominal Surgery | 99% | 0.92 (0.75-1.13) | 0.419 |
| Weight Difference 45-70kg (Donor – Recipient) | 75% | 0.52 (0.27-1.01) | 0.052 |
| Weight Difference >75kg (Donor – Recipient) | 75% | 0.82 (0.60-1.11) | 0.201 |
| Wait Time 0.5-1 Years | 94% | 1.01 (0.82-1.25) | 0.925 |


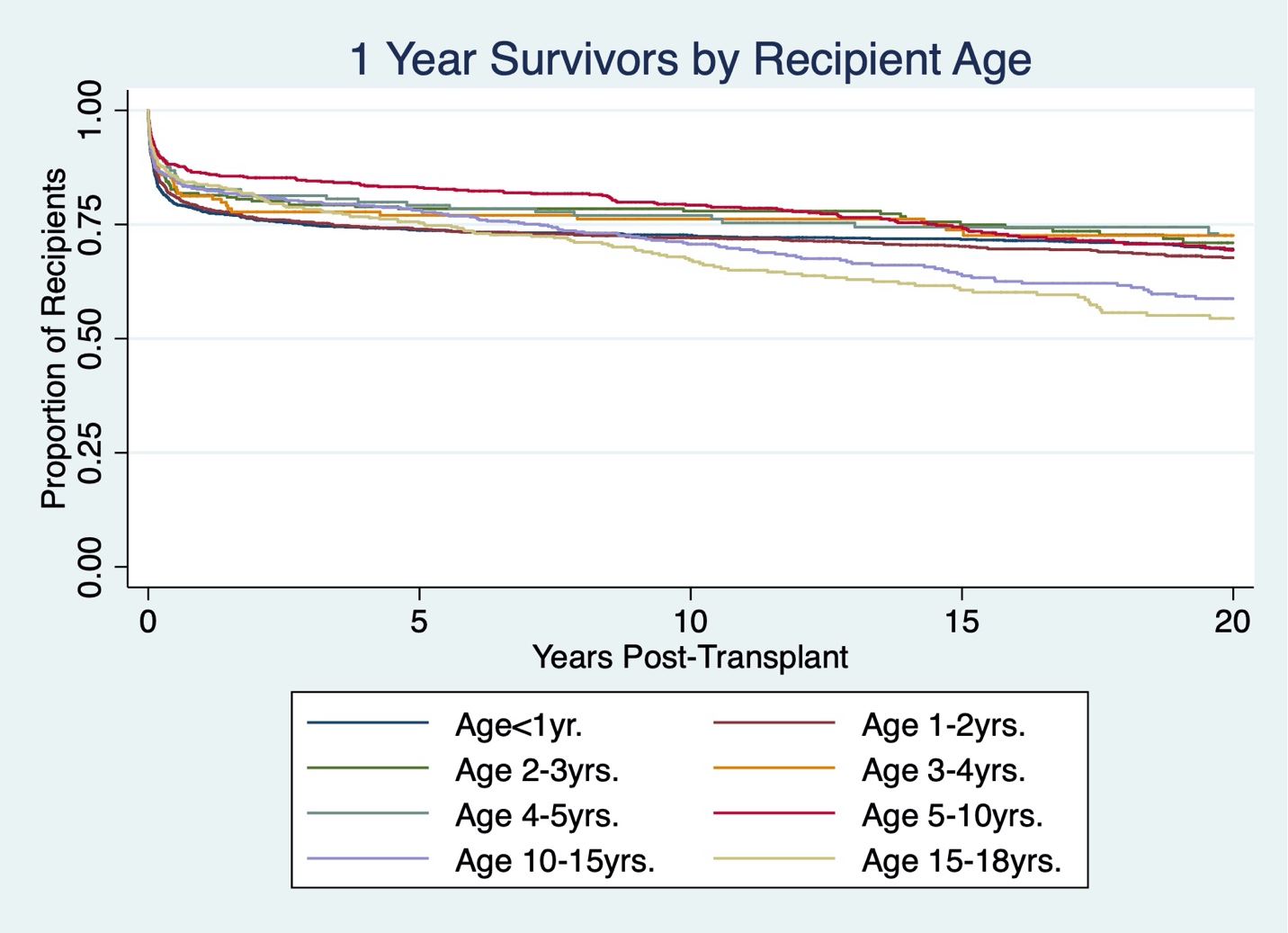


**Figure S1.** Kaplan-Meier Survival Function over time for All Survivors in Pediatric Liver Transplant recipients by recipient age
